# MODIFIED HEIDELBERG TECHNIQUE FOR PANCREATIC ANASTOMOSIS

**DOI:** 10.1590/0102-6720201700040008

**Published:** 2017

**Authors:** Orlando Jorge M TORRES, Roberto C N da Cunha COSTA, Felipe F Macatrão COSTA, Romerito Fonseca NEIVA, Tarik Soares SULEIMAN, Yglésio L Moyses S SOUZA, Shailesh V SHRIKHANDE

**Affiliations:** 1 Department of Gastrointestinal Surgery, Hepatopancreatobiliary Unit, Federal University of Maranhão, São Luiz, MA, Brazil;; 2 Department of Surgical Oncology, Tata Memorial Centre Hospital, Mumbai, India.

**Keywords:** Pancreatoduodenectomy, Pancreatic anastomosis, Surgical technique., Duodenopancreatectomia, Anastomose pancreática, Técnica cirúrgica

## Abstract

*****Background*** ****:**

Pancreatic fistula is a major cause of morbidity and mortality after pancreatoduodenectomy. To prevent this complication, many technical procedures have been described.

***Aim*:**

To present a novel technique based on slight modifications of the original Heidelberg technique, as new pancreatojejunostomy technique for reconstruction of pancreatic stump after pancreatoduodenectomy and present initial results.

***Method*:**

The technique was used for patients with soft or hard pancreas and with duct size smaller or larger than 3 mm. The stitches are performed with 5-0 double needle prolene at the 2 o’clock, 4 o’clock, 6 o’clock, 8 o’clock, 10 o’clock, and 12 o’clock, positions, full thickness of the parenchyma. A running suture is performed with 4-0 single needle prolene on the posterior and anterior aspect the pancreatic parenchyma with the jejunal seromuscular layer. A plastic stent, 20 cm long, is inserted into the pancreatic duct and extended into the jejunal lumen. Two previously placed hemostatic sutures on the superior and inferior edges of the remnant pancreatic stump are passed in the jejunal seromuscular layer and tied.

*****Results*** ****:**

Seventeen patients underwent pancreatojejunostomy after pancreatoduodenectomy for different causes. None developed grade B or C pancreatic fistula. Biochemical leak according to the new definition (International Study Group on Pancreatic Surgery) was observed in four patients (23.5%). No mortality was observed.

*****Conclusion*** ****:**

Early results of this technique confirm that it is simple, reliable, easy to perform, and easy to learn. This technique is useful to reduce the incidence of pancreatic fistula after pancreatoduodenectomy.

## RESUMO

***Racional*:**

Fístula pancreática é a principal causa de morbidade e mortalidade após duodenopancreatectomia. Muitos procedimentos técnicos têm sido descritos para prevenir esta complicação.

***Objetivo*:**

Apresentar uma nova técnica baseada em pequenas modificações sobre a técnica original de Heidelberg para pancreatojejunostomia na reconstrução do coto pancreático após duodenopancreatectomia e apresentar os resultados iniciais.

***Método*:**

Esta técnica foi utilizada para pacientes com pancreas de consistência firme ou amolecida e ducto pancreático maior ou menor que 3 mm. Os pontos são realizados com prolene 5-0, duas agulhas nas posições de 2, 4, 6, 8, 10 e 12 h, com espessura total no parênquima pancreático. Uma sutura continua é realizada com prolene 4-0 de agulha única na parede posterior e anterior do pâncreas com a camada seromuscular do jejuno. Uma sonda de silicone de 20 cm de comprimento é inserida no ducto pancreático em direção ao jejuno. As duas suturas hemostáticas previamente colocadas nas bordas superior e inferior do remanescente pancreático são passadas e amarradas com a camada seromuscular do jejuno.

Dezessete pacientes foram submetidos a pancreatojejunostomia após duodenopancreatectomia por diferentes causas. Nenhum desenvolveu fístula pancreática graus B ou C. Vazamento bioquímico, de acordo com a nova definição do Grupo Internacional de Estudo em Cirurgia do Pâncreas (ISGPS), foi observado em quatro pacientes (23,5%). Não houve mortalidade.

***Conclusão*:**

Os resultados iniciais desta técnica confirmam que é simples, confiável, fácil de realizar e de aprender. Ela é útil para reduzir a incidência de fistula pancreática após duodenopancreatectomia.

## INTRODUCTION

Pancreatic fistula is a frustrating complication and remains a major cause of morbidity and mortality after pancreatoduodenectomy. The current literature reports that the incidence of this complication ranges between 3-45%, with a high associated mortality rate. To prevent pancreatic fistula, various technical procedures have been described, including invagination technique, duct-to-mucosa, Blumgart technique, Peng technique, and modifications. The desirable one should be associated with a low rate of pancreatic fistula and ease to perform regardless of the texture of the pancreas and the duct diameter^1^ ^,^ ^6^ ^,^ ^9^ ^,^ ^13^ ^,^ ^14^. 

Technical aspects of the pancreatic anastomosis contribute to increasing the anastomotic failure rate, particularly in patients with soft pancreas and small pancreatic duct. Shrikhande and the Heidelberg group published 10 years ago an interesting technique of pancreatic anastomosis after pancreaticoduodenectomy^11^ ^,^ ^12^ ^,^ ^16^. 

The objective of this study was to introduce a small modification of the Heidelberg pancreatojejunostomy technique for reconstruction of the pancreatic stump after pancreatoduodenectomy that can be used in patients with soft or hard pancreas and with duct size smaller or larger than 3 mm.

## METHOD

### Technique

#### *Transection of the pancreas*

**FIGURE 1:**
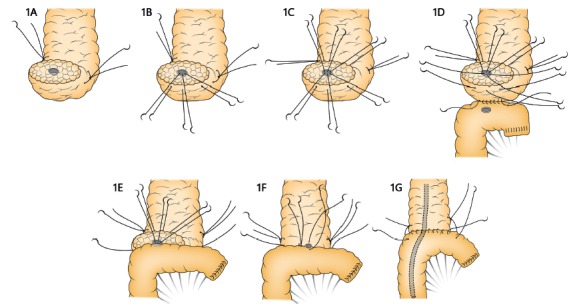
A) Cut surface of the pancreas, stay sutures and pancreatic duct; B) three stitches full thickness posterior at 4 o’clock, 6 o’clock, and 8 o’clock positions; C) three stitches full thickness anterior at 10 o’clock, 12 o’clock, and 2 o’clock positions; D) running suture on the posterior aspect; E) sutures are passed in the positions 4 o’clock, 6 o’clock, and 8 o’clock; F) sutures are passed in the positions 10 o’clock, 12 o’clock, and 2 o’clock and one stent is put into the pancreatic duct; G) running anterior suture and stay stitches.

Two stay sutures (Prolene 4-0, Ethicon^®^) are placed on both the superior and inferior margins of the pancreatic remnant (hemostatic sutures). The pancreatic parenchyma is then transected with a sharp knife, and hemostasis is performed with electrocautery (Figure 1A). After the resection phase by subtotal stomach-preserving pancreatoduodenectomy, the technique is completed and the specimen is retrieved. Then, a jejunal single limb is moved to the pancreatic cut end by a trans-mesocolic route and prepared for the anastomosis^15^. 

#### *Mobilization of the pancreatic remnant*

After the main pancreatic duct is identified, the cut surface of the pancreatic remnant is mobilized for approximately 1.5-2 cm of the length away from the splenic vein to allow the anastomosis. Small vessel branches are dissected and ligated (Figure 1A).

#### *Posterior duct-pancreatic suture*

Three sutures are placed on the posterior wall of the pancreatic duct to the posterior pancreatic parenchyma. The stitches are performed with 5-0 double needle prolene (Ethicon^®^) at the 4 o’clock, 6 o’clock, and 8 o’clock positions. The suture starts into the pancreatic duct traversing the full thickness of the parenchyma, until the posterior wall of the pancreas (from inside to outside). The free margin of the suture is at least 1 cm (Figure 1B).

#### *Anterior duct-pancreatic suture*

When all the posterior stitches are placed, three sutures are passed on the anterior wall of the pancreatic duct. The suture starts into the pancreatic duct, traversing the full thickness of the parenchyma, to the anterior wall of the pancreas (from inside to outside), similar to the posterior wall, at the 10 o’clock, 12 o’clock, and 2 o’clock positions. The posterior and anterior duct-pancreatic sutures are not knotted at this point (Figure 1C). 

#### *Posterior outer layer*

In this step, the anterior and posterior duct-pancreatic sutures are suspended with a Kelly clamp. The pancreatic stump and the anti-mesenteric border of the jejunum are located face to face. Is performed a running suture with 4-0 single needle prolene (Ethicon^®^) on the posterior aspect the pancreatic parenchyma with the jejunal seromuscular layer, beginning at the lower edge of the pancreas toward the upper edge (Figure 1D). 

#### *Posterior inner layer*

The jejunum is now opened on the anti-mesenteric side by electrocautery. The length of the enterotomy is approximately 0.5 cm long, near the pancreatic duct. The sutures in the 4 o’clock, 6 o’clock, and 8 o’clock positions (posterior duct-pancreatic suture) are passed from outside to inside in the inferior edge of the jejunum at the same positions (full thickness of the jejunum). The sutures are knotted at this time (Figure 1E). 

#### *Stent into the pancreatic duct*

A plastic stent, 20 cm long, is inserted into the pancreatic duct and, if necessary, secured with absorbable suture at the opening of the pancreatic duct. Approximately 15 cm of the length of the stent is extended into the jejunal lumen.

#### *Anterior inner layer*

The sutures in the 10 o’clock, 12 o’clock, and 2 o’clock positions (anterior duct-pancreatic suture) are passed from inside to outside in the superior edge of the jejunum (full thickness of the jejunum) and are knotted with the plastic stent into the jejunal lumen (Figure 1F). 

#### *Anterior outer layer*

A running suture is performed with 4-0 single needle prolene (Ethicon^®^), on the anterior aspect of the pancreatic parenchyma with jejunal seromuscular layer, beginning at the upper edge of the pancreas toward the lower edge (Figure 1G). 

#### *Stay suture with the jejunum*

Finally, the two previously placed hemostatic sutures on the superior and inferior edges of the remnant pancreatic stump are passed in the jejunal seromuscular layer and tied. These additional interrupted sutures placed between the pancreatic tissue and the jejunum must reinforce the anastomosis. 

Hepaticojejunostomy is performed with interrupted absorbable suture and gastrojejunostomy is performed by antecolic route, approximately 50 cm proximal to the hepaticojejunostomy. All patients have two drains placed near the pancreatico-jejunal anastomosis during the surgery. 

## RESULTS

In the period from July 2016 to June 2017, a total of 17 patients underwent pancreatoduodenectomy with this technique. The characteristics of the patients are presented in Table 1. There were 13 women (76.5%), and the median patient age was 54.1 years (20-76). Ductal adenocarcinoma was observed in nine patients (52.9%); soft texture of the pancreas was present in six (35.2%); duct size larger than 3 mm was found in nine (52.9%). Increased amylase in abdominal drainage (biochemical leak) was identified in four patients (23.5%), and resolved spontaneously within a week. Grade B or C fistula was not observed. No mortality occurred in this series.

**TABLE 1:** Characteristics of the patients

Characteristics	1	2	3	4	5	6	7	8	9	10	11	12	13	14	15	16	17
Age	61	47	28	40	70	20	59	60	70	60	56	36	76	38	70	62	67
Gender	M	M	F	F	M	M	F	F	F	F	F	F	F	F	F	F	F
Diagnosis	AV	AD	FT	AD	AD	NE	AD	IP	AD	AD	AD	AV	NE	AD	CC	AD	NE
Pancreas text	S	F	S	F	F	S	F	F	F	F	F	S	S	F	F	F	S
Duct size (mm)	≤3	≤3	≤3	>3	>3	≤3	>3	>3	≤3	≤3	>3	≤3	>3	>3	>3	>3	≤3
Op. time (min)	315	484	310	499	393	590	485	343	395	400	355	406	475	340	380	350	365
Transfusion	Y	N	Y	Y	N	N	N	N	Y	Y	N	N	Y	N	N	N	N
UCI time (d)	19	2	8	4	3	4	6	4	4	5	5	4	10	4	5	2	3
Fistula grade	A	-	A	-	-	A	A	-	-	-	-	-	-	-	-	-	-
LoS (d)	37	10	13	11	9	13	12	8	16	12	19	14	20	13	14	7	8
Mortality	N	N	N	N	N	N	N	N	N	N	N	N	N	N	N	N	N

Op=operative; ICU=Intensive Care Unit; LoS=length of stay; AD= adenocarcinoma; PA= hepatoduodenal ampulla tumor; CC=distal cholangiocarcinoma; FT=Frantz tumor; NE=neuroendocrine tumor; IP=IPMN; text=texture; S= soft; F=firm; Y=yes; N=no; d=days

## DISCUSSION

Pancreatic fistula is one of the most severe complications after pancreatoduodenectomy. The incidence is 3-45%, and results in a high rate of related mortality. To avoid this life-threatening complication, several techniques of pancreatoenteric anastomosis have been described, and the two primary methods are the duct-to-mucosa technique and the invagination technique. The procedures can be performed with the stomach or jejunum. Although some studies suggest that pancreaticogastrostomy is superior to pancreatojejunostomy, the ISGPS recommends pancreatogastrostomy or pancreatojejunostomy for the pancreaticoenteric anastomosis^1^ ^,^ ^2^ ^,^ ^6^ ^,^ ^8^. 

Many factors have been associated with failure of the pancreatoenteric anastomosis, including general factors, pathological factors, and technical factors. The most important risk factors related to pancreatic fistula are the diameter of pancreatic duct of 3 mm or less and the soft texture of the pancreas. In these situations, the type of pancreatoenteric anastomosis is important to reduce pancreatic fistula^9^ ^,^ ^10^. 

The ideal pancreatoenteric anastomosis should have the following characteristics: a) good blood supply to the pancreatic stump; b) pancreatic juice flow into the intestinal or gastric lumen; c) suitable for all pancreatic stumps and all pancreatic ducts; d) easy to perform and easy to learn^1^ ^,^ ^3^ ^,^ ^9^. 

Was described a novel technique for pancreatic anastomosis after pancreatoduodenectomy. The pancreatoduodenectomies were all performed by a team of surgeons who were trained in the performance of the pancreatoenteric anastomosis by the senior surgeon (OJMT). The novel pancreaticojejunostomy presented in this study differs slightly from the original technique published by Shrikhande et al. ^11^, and low rates of pancreatic fistula have been observed with the original technique^11^. 

According to the ISGPS’s new definition and grading of postoperative pancreatic fistula, grade A pancreatic fistula is now biochemical leak^1^. In the present study with the first unselected, consecutive series, none of patients developed grade B or C pancreatic fistula, and biochemical leak was observed in four patients (23.5%) after this technique. 

The series presented in this study demonstrates low rates of pancreatic fistula in comparison with other recent technical modifications in the literature (Table 2) ^3,5,7^. 

**TABLE 2:** Results of other technical modifications

Author	Fistula	Grade A	Grade B	Grade C	Mortality
Kim et al^7^	37,1%	17.9%	15.2%	4.0%	4.6%
Grobmyer et al^5^	20.3%	13.4%	3.7%	3.2%	1.6%
Chen et al^3^	24.5%	18.9%	5.6%	0.0%	0.0%

In the current study, was analyzed the drain amylase, which was elevated in 23.5% of the patients (biochemical leak) according to the new ISGPS definition^1^. Risk factors such as pancreatic duct size of 3 mm or smaller and soft texture of the pancreatic parenchyma were observed in the present series, but the same technique was suitable even for these patients.

In this technique, the six interrupted stitches, anterior and posterior full thicknesses, involve more pancreatic tissue. In addition, the stump is covered with the jejunum, reducing the risk of rupture. The length of the jejunostomy is approximately 0.5 cm long, smaller than that described by Shrikhande et al. ^11^, and is adjustable based on the diameter of the pancreatic duct. In addition, was inserted a plastic stent into the pancreatic duct to achieve better internal drainage from the pancreas to the intestinal lumen. Stents are not used in the original technique, and the diameter depends on the size of the duct. This procedure avoids or reduces pancreatic juice and bile retention in the initial segment of the jejunum when the peristaltic function is not restored. Furthermore, the procedure decreases the incidence of stricture formation around the pancreatic duct and reduces the possibility of inadvertent pancreatic duct occlusion. There is no need to remove the internal drainage stent. At the end of the procedure, after the anastomosis is completed, the stay suture is used to anchor the gland to the jejunum. This modification reduces pressure on the anastomosis suture line^11^. 

In the classical “duct-to-mucosa” anastomosis, the sutures involve a small portion of the pancreatic duct and the mucosa of the jejunum, but the pancreatic tissue is not included. The risk to tear and cause anastomotic rupture is relatively high^5^. In invagination pancreatojejunostomy, part of the pancreatic stump is invaginated into the lumen of the jejunum. The stitches are placed in the pancreatic parenchyma and capsule, but not in the pancreatic duct with the risk of laceration on tying. The pancreatic surface is exposed to the intestinal or gastric lumen, and hemorrhagic complications can occur. Furthermore, this technique is not suitable for patients with a large pancreatic stump^4^ ^,^ ^9^. 

Our modification of the original technique described by Shrikhande et al^11^. seems to be safe for soft or hard pancreas and any size of pancreatic duct. The small number of patients with this new pancreatoenteric anastomosis is the limitation of this study, and others are necessary to assess the utility of this procedure in decreasing postoperative fistula rates. 

## CONCLUSION

Early results of this technique confirm that it is simple, reliable, easy to perform, and easy to learn. This technique is useful to reduce the incidence of pancreatic fistula after pancreatoduodenectomy. 

## References

[1] Bassi C, Marchegiani G, Dervenis C, Sarr M, Abu-Hilal M, Adam M (2016). The 2016 update of the International Study Group (ISGPS)
definition and grading of postoperative pancreatic fistula: 11 Years
After. Surgery.

[2] Callery MP, Pratt WB, Kent TS, Chaikof EL, Vollmer CM (2013). A prospectively validated clinical risk score accurately predicts
pancreatic fistula after pancreatoduodenectomy. J Am Coll Surg.

[3] Chen Y, Zhu X, Huang J, Zhu Y (2015). End-to-side penetrating-suture pancreaticojejunostomy A novel
anastomosis technique. J Am Coll Surg.

[4] Giudici F, Pesi B, Zambonin D, Scaringi S, Bechi P, Batignani G (2016). Safer intestinal invagination for a solid pancreatico-jejunal
anastomosis in presence of a soft texture pancreatic remnant and non-dilated
duct. Hepatobiliary Pancreat Dis Int.

[5] Grobmyer SR, Kooby D, Blumgart LH, Hochwald SN (2010). Novel pancreaticojejunostomy with a low rate of anastomotic
failure-related complications. J Am Coll Surg.

[6] Hackert T, Werner J, Buchler MW (2011). Postoperative pancreatic fistula. The Surgeon.

[7] Kim M, Shin WY, Lee KY, Ahn SI (2017). An intuitive method of duct-to-mucosa pancreaticojejunostomy
after pancreaticoduodenectomy use of one-step circumferential interrupted
sutures. Ann Hepatobiliary Pancreat Surg.

[8] Liu FB, Chen JM, Geng W, Xie SX, Zhao YJ, Yu LQ, Geng XP (2015). Pancreaticogastrostomy is associated with significantly less
pancreatic fistula than pancreaticojejunostomy reconstruction after
pancreaticoduodenectomy a meta-analysis of seven randomized controlled
trials. HPB.

[9] Schoellhammer HF, Fong Y, Gagandeep S (2014). Techniques for prevention of pancreatic leak after
pancreatectomy. Hepatobiliary Surg Nutr.

[10] Shrikhande SV, Barreto SG, Shukla PJ (2008). Pancreatic fistula after pancreaticoduodenectomy The impact of a
standardized technique of pancreaticojejunostomy. Langenbecks Arch Surg.

[11] Shrikhande SV, Kleeff J, Büchler MW, Friess H (2007). Pancreatic anastomosis after pancreaticoduodenectomy how we do
it. Indian J. Surg.

[12] Shrikhande SV, Sivasanker M, Vollmer CM, Friess H, Besselink MG, Fingerhut A (2017). Pancreatic anastomosis after pancreatoduodenectomy a position
statement by the International Study Group of Pancreatic Surgery
(ISGPS). Surgery.

[13] Torres OJM, Barbosa ES, Barros NDC, Barros CA, Ferreira EDZ, Pereira HC (2007). Pancreaticoduodenectomies analysis of 39 patients. Rev Col Bras Cir.

[14] Torres OJM, Fernandes ESM, Vasques RR, Waechter FL, Amaral PCG, Rezende MB, Montenegro-Costa R, Montagnini AL (2017). Pancreatoduodenectomy Brazilian practice patterns. Arq Bras Cir Dig.

[15] Torres OJM, Vasques RR, Torres CCS (2016). The obituary of the pylorus-preserving
pancreatoduodenectomy. Arq Bras Cir Dig.

[16] Z'graggen K, Uhl W, Friess H, Büchler MW (2002). How to do a safe pancreatic anastomosis. J Hepatobiliary Pancreat Surg.

